# Prognostic factors affecting local control of hepatic tumors treated by stereotactic body radiation therapy

**DOI:** 10.1186/1748-717X-7-166

**Published:** 2012-10-10

**Authors:** Sylvain Dewas, Jean-Emmanuel Bibault, Xavier Mirabel, Ingrid Fumagalli, Andrew Kramar, Hajer Jarraya, Thomas Lacornerie, Claire Dewas-Vautravers, Eric Lartigau

**Affiliations:** 1 Academic Radiation Oncology Department & University Lille II, CLCC Oscar Lambret, 3 rue Frederic Combemale, BP 307, Lille cedex, 59 020, France; 2 Methodology and Biostatistics Unit, CLCC Oscar Lambret, 3 rue Frederic Combemale, BP 307, Lille cedex, 59 020, France; 3 Department of Radiology, CLCC Oscar Lambret, 3 rue Frederic Combemale, BP 307, Lille cedex, 59 020, France

**Keywords:** Hepatocellular carcinoma, Liver metastases, SBRT, Local control, Prognostic fractors

## Abstract

**Purpose:**

Robotic Stereotactic Body Radiation Therapy with real-time tumor tracking has shown encouraging results for hepatic tumors with good efficacy and low toxicity. We studied the factors associated with local control of primary or secondary hepatic lesions post-SBRT.

**Methods and materials:**

Since 2007, 153 stereotactic liver treatments were administered to 120 patients using the CyberKnife® System. Ninety-nine liver metastases (72 patients), 48 hepatocellular carcinomas (42 patients), and six cholangiocarcinomas were treated. On average, three to four sessions were delivered over 12 days. Twenty-seven to 45 Gy was prescribed to the 80% isodose line. Margins consisted of 5 to 10 mm for clinical target volume (CTV) and 3 mm for planning target volume (PTV).

**Results:**

Median size was 33 mm (range, 5–112 mm). Median gross tumor volume (GTV) was 32.38 cm^3^ (range, 0.2–499.5 cm^3^). Median total dose was 45 Gy in three fractions. Median minimum dose was 27 Gy in three fractions. With a median follow-up of 15.0 months, local control rates at one and two years were 84% and 74.6%, respectively. The factors associated with better local control were lesion size < 50 mm (p = 0.019), GTV volume (p < 0.05), PTV volume (p < 0.01) and two treatment factors: a total dose of 45 Gy and a dose–per-fraction of 15 Gy (p = 0.019).

**Conclusions:**

Dose, tumor diameter and volume are prognostic factors for local control when a stereotactic radiation therapy for hepatic lesions is considered. These results should be considered in order to obtain a maximum therapeutic efficacy.

## Introduction

Stereotactic body radiation therapy (SBRT) for hepatic metastases (HM) and hepatocellular carcinoma (HCC) has shown encouraging rates of local control and low toxicity [1-4]. Many patients with primary or secondary hepatic lesions can now be treated with these new techniques. However relevant data and studies are still required to define which patients will benefit best from it and which fractionation regimen and total dose should be used. Unfortunately, the published series are heterogeneous for the doses used as well as the number of patients treated and do not allow for reliable analysis. Since July 2007, a CyberKnife® System (Accuray Incorporated, Sunnyvale, California, U.S.A.) has been available in our department. This image-guided system delivers hypofractionated robotic SBRT. Herein, we present our results for 153 primary or secondary hepatic lesions treated with SBRT and the factors associated with tumor control.

## Materials and methods

### Patients

Between July 2007 and March 2010, 120 patients underwent SBRT with real-time tracking for primary or secondary hepatic lesions. Seventy-two patients were treated for HM, 42 for HCC (36 patients with A and 6 patients with B Child-Pugh score) and six for cholangiocarcinoma (CC).

Patients with HM were ineligible for hepatic surgery or radiofrequency ablation. Other selection criteria for HM were WHO score under 3, four or less hepatic lesions and lesion size under 100 mm. For HCC, selection criteria were WHO score under 3, two or less lesions, Child-Pughs score A or B. For cholangiocarcinoma, selection criteria were WHO score under 2 and target size under 120 mm. SBRT is considered for patients for whom standard treatments such as resection, transplantation, chemoembolization or radiofrequency ablation were not feasible. Informed consent was obtained in accordance with the guidelines of the French National Cancer Institute (Institut National du Cancer) required when assessing the efficacy and toxicity of a novel therapy,

All treatments were delivered with the CyberKnife system, at the Academic Department of Radiotherapy, Centre Oscar Lambret, Lille. The population treated by SBRT for hepatic tumors consisted of 120 patients (78 men and 42 women). The average age was 65 years (range, 23 to 85 years). There were a total of 153 tumors; 97 patients had one, 15 patients two, six patients three, and two patients had four tumors treated. The median diameter of a tumor was 33 mm (range, 5 to 112 mm). The median volume of GTV was 32.38 cm^3^ (range, 0.2 to 499.5 cm^3^).

### Planning and treatment

The outpatient treatments were conducted using the Multiplan® v3 treatment planning software (Accuray) and the Synchrony® Respiratory Tracking System (Accuray), enabling the tracking of tumor movement in real time. Gold markers (fiducials) were implanted around the lesion before the planning CT. Three to four fiducials are implanted close to the target by a radiologist with a CT scan while the patient is under local anesthesia. The median time between fiducial implantation and the beginning of the treatment was 29 days (minimum seven days). A triphasic (arterial phase, portal phase, and late phase) planning CT scan was recorded twice with 50 cc of iodinated contrast agent, 1- and 3-mm slice thickness with the patient lying on a vacuum mattress or a self-expanding foam mattress. Gross Tumor Volume (GTV) was based on the better of either the contrast-enhanced planning CT or MRI, depending on image quality. Clinical Target Volume (CTV) was 5 mm geometric expansion of the GTV (CTV = GTV + 5 mm) to treat any microscopic disease extension. The margin was 1 cm within the liver, and none outside, in cases of HCC and CC. Planning Target Volume (PTV) contained the CTV and a 3-mm geometric margin to account for the uncertainty secondary to the slice thickness. Two weeks were usually necessary between dosimetric CT and start of treatment. The prescribed dose was 45 Gy for HCC. Early HM cases received 40 Gy in four equal fractions; then the dose was increased to 45 Gy in three equal fractions, as in the cases of HCC. Dose constraints are shown in Table 1. Doses slightly varied based on the location of the tumor and the nearby organs at risk. Normal tissue constraints used for liver lesions included the volume receiving 21 Gy (V21 < 33%) < 33% and V15 < 50% for normal liver. If this constraint was not possible, dose was decreased. The dose was prescribed to the 80% isodose line (95% of the PTV receiving the total dose) and delivered in an average of eight days (range, four to 17 days).

**Table 1:** Dose constraints (for 3 to 4 fractions)

**Organ**	**Dose constraints**
Liver	V21 < 33%
Spinal cord	Dmax < 22 Gy
Kidney	V15 < 33%
Stomach	V21 < 5 cm^3^
Duodenum	V15 < 5 cm^3^
	Dmax < 24 Gy
Small intestine	V16 < 5 cm^3^
	Dmax < 27 Gy

### Follow-up

All patients had a contrast-enhanced CT scan of the thorax, abdomen, and pelvis, and a hepatic MRI when it was available, at the time of treatment. Imaging was repeated at each follow-up every three months in addition to the clinical examinations and biochemical tests. Evaluations took into account changes in tumor vascularization, as recommended by EASL (European Association for the Study of the Liver) [5]. Local response was considered complete in case of disappearance of the target lesion or its replacement by fibrotic scar not changing in size or partial when at least a 30% decrease in the largest diameter of the target was observed. Failure was defined as an increase of at least 20% in the size of the largest axis of the target. Absence of any of the above was considered stable disease including any changes in vascularization determined by contrast intake. Local control was imaging-based absence of progression. Follow-up was defined has the time between last treatment session and last evaluation.

### Statistics

The software STATA v11 (Stata Corporation, College Station, Texas, USA) was used for the statistical analyses. Time to local failure was defined from the beginning of the treatment (as opposed to first diagnosis) until progression. Rates were estimated using the Kaplan-Meier method. Differences among survival curves were compared using the log-rank test. The univariate analyses of local control were performed using the Cox regression model. Fisher exact test and Pearson chi-squares methods were used to study the association between categorical variables, and the Kruskal-Wallis test for continuous variables. The Pearson correlation coefficient was used to assess the association between two quantitative variables. A p value <0.05 was chosen as the significance threshold.

## Results

### Patient and treatment characteristics

Patient characteristics are shown in Table 2. Median tumor diameter was 33 mm (range, 5 to 112 mm); median GTV was 32.38 cm^3^ (range, 0.2 to 499.5 cm^3^) and median hepatic volume was 1,606 cm^3^ (range, 843 to 2,940 cm^3^). Dosimetric and technical data are presented in Table 3. Mean session length was 107 minutes (range, 36 to 199 minutes); average number of beams per treatment was 152 (range, 25 to 276 beams); median total dose was 45 Gy (15 Gy per session); the lowest total dose was 27 Gy (9 Gy per session); median follow-up was 15.0 months (CI 95%: 12.4 to 17.5 months).

**Table 2:** Patient characteristics

	**Total N (%) or Median (range)**	**Hepatocarcinoma**	**Hepatic metastases**	**Cholangiocarcinoma**	p*
**Sex**					
**Male**	**78 (65%)**	32 (76.2%)	42 (58.3%)	4 (66.7%)	**0.14**
**Female**	**42 (35%)**	10 (23.8%)	30 (41.7%)	2 (33.3%)	
**Age**	**67 (23--85)**	69 (43--85)	64 (23--83)	70 (62--78)	**0.16**
**Weight (kg):**	**78 (43--120)**	83 (47--120)	74.5 (43--115)	73 (68--85)	**0.07**
**BMI**	**26.1 (14.2--43.3)**	29 (20.5--40.6)	25.6 (14.2--43.3)	27.1 (23.5--32)	**0.03**
**WHO**					
**0**	**92 (77.3%)**	31 (73.8%)	58 (81.7%)	3 (50%)	
**1**	**23 (18.5%)**	11 (26.2%)	9 (12.7%)	2 (33.3%)	**0.08**
**2**	**4 (3.4%)**	0	3 (4.2%)	1 (16.7%)	
**3**	**1 (0.8%)**	0	1 (1.4%)	0	
**Prior treatments**	**114 (95%)**	19 (45.2%)	66 (91.7%)	3 (50%)	**< 0.001**
**Radiofrequency**	**11 (9.2%)**	2 (4.8%)	9 (12.5%)	0	**0.38**
**Surgery**	**31 (25.8%)**	4 (9.5%)	24 (33.3%)	3 (50%)	**0.003**
**Chemo--embo**	**8 (6.7%)**	6 (14.3%)	2 (2.8%)	0	**0.11**
**Radiotherapy**	**2 (1.7%)**	1 (2.4%)	1 (1.4%)	0	**0.87**
**Chemotherapy**	**62 (51.7%)**	6 (14.3%)	54 (75%)	2 (33.3%)	**< 0.001**
**Hepatic volume (cm**^**3**^**)**	**1548 (843–2940)**	1614 (908–2611)	1499 (843–2940)	1783 (1607–2018)	**0,19**
**Targets size (Sum in mm per patient)**	**45 (8–159)**	40(17–145)	45 (8–159)	63 (36–112)	**0,24**

*Chi-square/Fisher exact or Kruskal-Wallis.

**Table 3:** Treatment characteristics

	**Total N (%) or Median (range)**	**Hepatocarcinoma**	**Hepatic metastases**	**Cholangiocarcinoma**	p*
**Sum of GTV volumes (cm**^**3**^**)**	**32.9 (0.2--499)**	47.5 (1.4--499)	25.6 (0.2--245)	261 (8--371)	**0.029**
**Ratio of the volume of GTV to the Liver**	**0.02 (0--0.32)**	0.03 (0--0.3)	0.02 (0--0.2)	0.1 (0--0.2)	**0.028**
**Ratio of the volume of PTV to the Liver**	**0.08 (0--0.38)**	0.09 (0--0.38)	0.05 (0--0.37)	0.2 (0--0.3)	**0.074**
**Session length (min)**	**107 (35--199)**	107 (35--156)	107 (41--199)	100 (74--151)	**0.831**
**Number of beams (median)**	**150 (25--276)**	148 (29--254)	149 (25--276)	182 (105--226)	**0.449**
**Total dose (Gy)**	**45 (27--45)**	45 (27--45)	45 (27--45)	45 (29--45)	**0.321**
		-- 45 Gy: 15 Gy x 3	-- 45 Gy: 15 Gy x 3	-- 45 Gy: 15 Gy x 3	
		-- 39 Gy: 13 Gy x 3	-- 40 Gy: 10 Gy x 4	-- 40 Gy: 10 Gy x 4	
		-- 36 Gy: 12 Gy x 3	-- 39 Gy: 13 Gy x 3	-- 39 Gy: 13 Gy x 3	
		-- 30 Gy: 15 Gy x 2	-- 36 Gy: 12 Gy x 3		
		-- 30 Gy: 10 Gy x 3	-- 30 Gy: 10 Gy x 3		
		-- 27 Gy: 9 Gy x 3			
**Dose per fraction (Gy)**	**15 (9--15)**	15 (9--15)	15 (9--15)	15 (10--15)	**0,026**

*Chi-square/Fisher exact or Kruskal-Wallis.

### Local control

**Figure 1:**
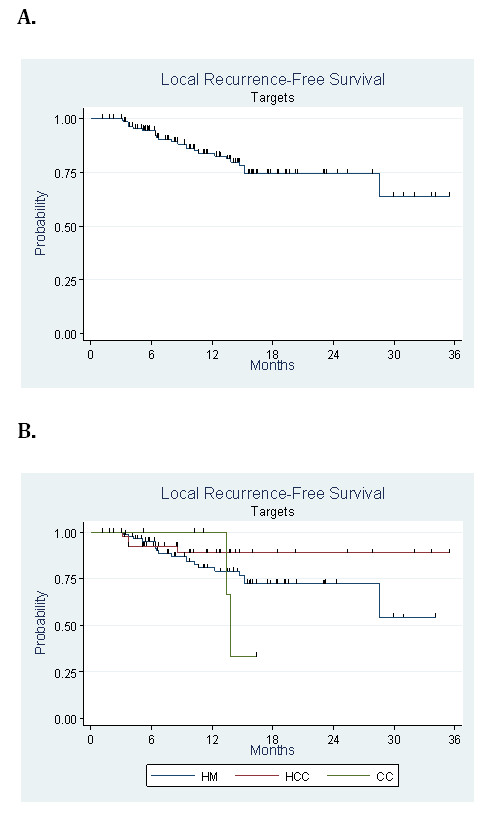
Local recurrence-free survival by target (A) (n = 153) and according to type of lesion (B).

Twenty-two patients showed progression of one or more treated lesions (18.3%). Three patients with HCC (7.3% of HCC cohort), 17 patients with HM (25.4% of HM cohort) and two patients with cholangiocarcinoma (33.3% of CC cohort) experienced failures. Median time to recurrence for HCC patients was 3.71 months, compared to 6.74 months for HM and 13.6 months for CC (p = 0.075). Overall one and two-year local control rates regardless of pathology were 80.4% (CI 95%: 70.1% to 87.5%) and 72.5% (CI 95%: 60.2 to 81.6%) respectively. For HCC the estimated local control rates at one and two years were 90.5%. For HM, the one and two year local control rates were 73.3% and 67.4%, respectively. Based on the number of tumors, among the 153 lesions (99 MH, 48 HCC, and six CC tumors) four HCC tumors (8.7%), 19 HM tumors (20.2%), and two CC tumors (33.3%) progressed at a median 3.7 months, eight months, and 13.6 months, respectively (Figure 1). The local control rate regardless of pathology was 83.7%. Local control rates regardless of primary at one and two years were 84% (CI 95%: 75.6% to 89.7%) and 74.6% (CI 95%: 63.7% to 82.7%), respectively. For HCC, the rate was 88.9% at both one and two years (CI 95%: 72.5 to 95.8%); for HM, these rates are 80.9% (CI 95%: 69.7 to 88.3%) at one year and 72.5% at two years (CI 95%: 59.3 to 82%). For CC, local control rate were 100% at one year and two-year follow-up was not reached. Median survival without local recurrence was not reached.

**Figure 2:**
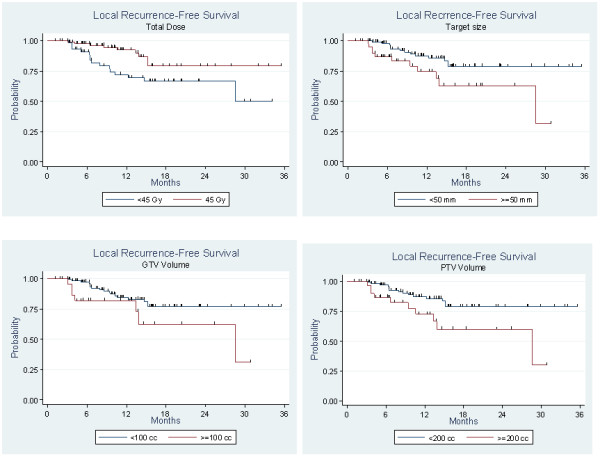
Local control per target. Factors associated with better local control: a dose of 45 Gy (p = 0.019), target diameter <50 mm (p = 0.019), a GTV <100 cc (p = 0.063) and a PTV <200 cc (p = 0.014).

**Figure 3:**
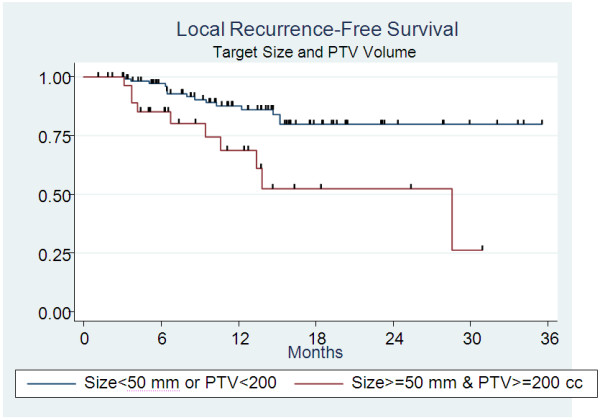
Local recurrence-free survival according to target diameter and PTV (p = 0.004).

We performed a univariate analysis to identify prognostic factors of local control : factors included in the univariate analysis were : target diameter, GTV, PTV, total dose, dose per fraction, number of fractions and session length. The results are presented in Table 4 and Figure 2. Accordingly, PTV appeared to be linked to local control Figure 3 (p = 0.014).

**Table 4:** Univariate analysis of local control at one and two years per target (n = 153)

	**Local control per target at 1 year (%)**	**Local control per target at 2 years (%)**	p*
**Total dose**			
**= 45 Gy**	92.3 (82.1 – 96.8)	79.1 (61.6 – 89.3)	
**< 45 Gy**	72.0 (56.4 – 82.8)	66.5 (50.3 – 78.5)	**0.019**
**Target diameter**			
**> 50 mm**	74.6 (54.6 – 86.8)	63.0 (39.8 – 79.3)	
**< 50 mm**	87.5 (77.9 – 93.1)	78.8 (65.9 – 87.3)	**0.019**
**GTV volumes**			
**> 100 cc**	81.8 (58.5 – 92.8)	62.3 (31.0 – 82.6)	
**< 100 cc**	84.4 (74.9 – 90.5)	76.8 (64.9 – 85.0)	**0.063**
**PTV volumes**			
**> 200 cc**	72.6 (50.3 – 86.2)	59.9 (35.3 – 77.7)	
**< 200 cc**	87.2 (78.0 – 92.8)	79.0 (66.7 – 87.2)	**0.014**

* Log-rank test (CI at 95%).

Median time to local recurrence in the target when the dose was less than 45 Gy, or when the dose per fraction was less than 15 Gy, was 28.6 months. Median time to recurrence with higher doses was not reached (p = 0.019). The recurrence rate for tumors with PTV >200 cc was 33.3% compared to 13% when the volume was smaller (p = 0.014).

## Discussion

### Hepatocellular carcinoma

We observed a local control rate at one and two years of 90.5%. In other series in the literature, rates of local control vary from 65% to 100% (Table 5). The local control rate is 100% for the four HCC and one CC in the study of Wulf et al. [6]. The study by Choi et al. found a local control of 71.9% at a median follow-up of 10.5 months [7]. SBRT for HCC has found a role and been studied as a local salvage treatment after incomplete transarterial chemoembolization [8], as a bridge towards transplantation [9] or for recurrence treatment [10]. Each study found SBRT to be both a feasible and promising treatment for patient without any other treatment option. 

**Table 5:** Published studies of stereotactic radiation therapy for hepatic lesions

***Study***	***No. of lesions***	***No. of patients***	***Total dose (Gy)***	***No. of fractions***	***Lesion volume/Lesion size (medians)***	***Post-treatment period median in months***	***Local control at 1 and 2 years***	***Comments***
Blomgren et al. 1998 [20]	HCC and CC (n = 20)	41	30	2-3	22 cc/-	11	100% overall control	Retrospective
	HM (n = 21)				24 cc/-		95% overall control	
Herfarth et al. 2001 [13]	HCC + CC (n = 4)	37	14-26	1	10 cc/-	-	67/-	Phase I/II
	HM (n = 56)							
Fuss et al. 2004 [21]	HCC (n = 1)	15	36	3-6	56 cc/-	6.5	94/-	Retrospective
	HM (n = 17)							
Schefter et al. 2005 (27)	HM (n = 18)	18	36-60	3	18 cc/-	-	-	Phase I
								Body Frame
Mendez et al. 2006 [22]	HCC (n = 11)	25	25–37.5	3-5	22 cc/32 mm	12.9 (1.1–322)	94/82	Phase I/II
								Body Frame
							75/75 (HCC)	
	HM (n = 34)						100/86	
							(HM)	
Wulf et al. 2006 [6]	HCC + CC (n = 5)	44	21-36	1-3	-	15	92/66	Phase I
								Body Frame
	HM (n = 39)							
Hoyer et al. 2006 [23]	HM CRC (n = 44)	64	45	3	-/35	4.5 years	-/79	Phase II
								Body Frame
Kavanagh et al. 2006 [24]	HM (n = 36)	36	60	3	14 cc/6	19	93% at 18 months	Phase I/II
								Body Frame
Katz et al. 2007 [25]	HM (n = 174)	69	30-55	7-20	9.9 cc/27 mm	14.5	76/57	Retrospective
								Exac Trac
Tse et al. 2008 [26]	HCC + CC (n = 31 + 10)	41	24–54	6	173 cc	17.6 (10.8 – 39.2)	65%	Phase I
								Respiration control
Choi et al. 2008 [7]	HCC (n = 32. including 9 PT)	31	30 – 39	3	25 cc/-	10.5 (2 – 18.5)	71.9% at the median of 10.5 months	Retrospective
								CyberKnife
Rusthoven et al. 2009 [15]	HM (n = 63)	47	60	3	15 cc/27 mm	16	95/92	Phase I/II
Lee et al. 2009 [27]	HM (n = 68)	68	27-60	6	75.2 cc/-	10.8	71/-	Phase I
								Respiration control
Ambrosino et al. 2009 [1]	HM (n = 27)	27	25-60	3	69 cc/-	13 (6–16)	85.2% overall control	Retrospective
								CyberKnife
Goodman et al. 2009 [3]	HCC + CC (n = 7)	26	18-30	1	33 cc/-	17.3 (2–55)	77/-	Phase I
								CyberKnife
	HM (n = 19)							
Van der Pool et al. 2010 [4]	HM CRC (n = 31)	20	37.5	3	-/23 mm	26 (6–57)	-/74	Retrospective
								Body Frame
Cardenes et al. 2010 [28]	HCC (n = 25. including 3 PT)	17	36-48	3	34 cc/40 mm	24 (10–42)	100%	Phase I
								CyberKnife
Present study 2010	153	120	27-45	2-4	73 cc/48 mm	15 (12–18)	80.4/72.5	Retrospective
								CyberKnife
	HCC (n = 48. including 3 PT)	42	27-45	2-3	87 cc/48 mm	13.7	90.5/90.5	
							73.3/67.4	
	HM (n = 99)	72	30-45	3-4	54 cc/47 mm	15.5	100/-	
	CC (n = 6)	6	39-45	3-4	208 cc/65 mm	11		

*HCC*: hepatocarcinoma, *HM*: hepatic metastases, *CC*: intrahepatic cholangiocarcinoma, *PT*: portal thrombosis, *CRC*: colorectal cancer.

### Cholangiocarcinoma

Data on cholangiocarcinoma treated with SBRT is scarce. A study published by Kopek et al. [11] reported the results of SBRT for 27 patients with unresectable cholangiocarcinoma. 45 Gy were administered in three fractions. With a median follow-up of 5.4 years, the median progression-free survival and overall survival were 6.7 and 10.6 months respectively. Another study found similar interesting results with a one-year local-control rate of 100% [12]. However these studies had a limited number of patients. Their results are comparable to the efficacy of fractionated chemoradiotherapy for these poor-prognosis patients. SBRT could however be easier and faster to perform with less toxicity for the patient.

### Metastases

Regarding metastatic hepatic lesions in the literature, the median follow-up ranges from six to 54 months. The local control rate varies between 82% and 100%. At one and two years, actuarial control rates range from 71% to 100% and from 64% to 86% The only prognostic factor found is the dose, tumors receiving a lower dose having a poorer local control.

### Total dose as a major prognostic factor

One of the first prospective studies using stereotactic radiotherapy as a single fraction with doses of 14–26 Gy for the treatment of hepatic tumors, including HCC and metastases, was conducted by Herfarth et al. [13]. The actuarial local control rates were 75%, 71%, and 67% at six-, 12-, and 18-months respectively. Updating these results yielded a local control rate of 66% at 18 months after a single fraction of 22 Gy [14]. The authors showed that the reduction in tumor volume may be delayed after a single irradiation dose; eight of the 17 patients with stable disease at the first evaluation achieved a complete or partial response at last follow-up. More recently, Ambrosino et al. published their preliminary results of SBRT by CyberKnife for unresectable hepatic lesions in 27 patients [1]. They obtained disease control in 74.1% of cases. The latest study to our knowledge was by Van der Pool et al. [4], reporting a local control rate of 74% at two years, with the Body Frame system, after three sessions of 12.5 Gy. Our results appear quite similar to these studies, local control rates at one and two years being 73.3% and 67.4%, respectively. The prescription dose is very heterogeneous in these studies, making comparison of the results difficult. The importance of the dose in the treatment of hepatic metastases has already been suggested. In the study by Herfarth, progression was observed in 12 of 55 tumors (22%) [13]. The difference in local tumor control between the group receiving 20 to 26 Gy, compared with the group receiving 14 to 20 Gy, was statistically significant (p <0.001). The diameter and the volume of the GTV and the PTV were identified in our series as prognostic factors of local control. The influence of the tumor size remains controversial in the literature. According to Rusthoven et al., size plays a role in local control: lesions less than 3 cm have a local control rate at two years of 100%, compared to 77% for lesions greater than 3 cm (p = 0.0015) [15]. In other series, stratification by size reveals no significant difference in local control between lesions larger than 15 cm^3^ compared with lesions less than 15 cm^3^ in patients treated with doses above 20 Gy [13].

In the literature, the treatment technique, dose, and fractionation used vary from one study to the other. The doses delivered range from 30 Gy in a single fraction to 54 Gy in six fractions of 9 Gy [6,7,16]. It has been demonstrated that there is a dose–response relationship for HCC with non-stereotactic conformal hepatic irradiation with an increased response rate as the dose increases [17,18]. The same relationship holds when reproduced using the milimetric tracking accuracy of CyberKnife. Our treatment regimen enables delivering a total dose of 45 Gy in three fractions of 15 Gy. According to the quadratic linear model, the equivalent of this dose in conventional fractionation of 2 Gy for an alpha/beta ratio of 10 Gy would be 112.5 Gy. Regarding fractionation, the regimens with a single session seem a little less effective in terms of local control, especially those using a low dose per fraction [13,17]. A recent study reviewed 141 patients treated with hepatic and pulmonary stereotactic radiation therapy, without distinction, in order to find predictive factors of local control [16]. There were 246 lesions (165 lung cancers and 81 patients with hepatic lesions). The factors found in that analysis were also, as in our series, the delivered dose (p <0.001) and a tendency for the GTV (p = 0.064) in multivariate analysis. In another study, the team of Wada et al. demonstrated a role for target size in 24 patients treated for 42 lesions in both lung and hepatic locations [19]. A lesion of less than 3 cm in diameter was associated with better local control (p = 0.0022). While many studies show the interest of SBRT for liver tumors [20-28], most of them are retrospective or with a limited number of patients. Dose regimens and delivery techniques vary to a great extent between each of them. A multicentric, randomized trial comparing SBRT to surgery could be of great interest in this context.

## Conclusion

Our experience including 120 patients, reported in this work, is to our knowledge the largest series in the literature. Factors limiting the effectiveness of this treatment presented in this work are: lesion diameter, the volume of the tumor and of the PTV. A total dose of 45 Gy and 15 Gy per fraction is necessary for maximum efficiency. There is a certain dose–response relationship for HCC, as well as with HM, prompting a high dose level as possible.

## Competing interests

Pr Eric Lartigau is a consultant for ACCURAY.

## Authors’ contributions

SD and XM conceived the study. SD collected data. SD and JEB drafted the manuscript. XM, IF, HJ, TL, CDV and EL participated in coordination and helped to draft the manuscript. AK performed the statistical analyses. EL provided mentorship and edited the manuscript. All authors have read and approved the final manuscript.

## References

[B1] Ambrosino G Polistina F Costantin G Image-guided robotic stereotactic radiosurgery for unresectable liver metastases: preliminary results Anticancer Res 2009 29 3381 3384 19661360

[B2] Cárdenes HR Role of stereotactic body radiotherapy in the management of primary hepatocellular carcinoma. Rationale, technique and results Clin Transl Oncol 2009 11 276 283 10.1007/s12094-009-0355-5 19451060

[B3] Goodman KA Wiegner EA Maturen KE Dose-escalation study of single-fraction stereotactic body radiotherapy for liver malignancies Int J Radiat Oncol Biol Phys 2010 78 486 493 10.1016/j.ijrobp.2009.08.020 20350791

[B4] van der Pool AEM Méndez Romero A Wunderink W Stereotactic body radiation therapy for colorectal liver metastases Br J Surg 2010 97 377 382 10.1002/bjs.6895 20095016

[B5] Bruix J Sherman M Llovet JM Clinical management of hepatocellular carcinoma. Conclusions of the Barcelona-2000 EASL conference. European Association for the Study of the Liver J Hepatol 2001 35 421 430 10.1016/S0168-8278(01)00130-1 11592607

[B6] Wulf J Hädinger U Oppitz U Stereotactic radiotherapy of targets in the lung and liver Strahlenther Onkol 2001 177 645 655 10.1007/PL00002379 11789403

[B7] Choi BO Choi IB Jang HS Stereotactic body radiation therapy with or without transarterial chemoembolization for patients with primary hepatocellular carcinoma: preliminary analysis BMC Cancer 2008 8 351 10.1186/1471-2407-8-351 19038025 PMC2615783

[B8] Kang J-K Kim M-S Cho CK Stereotactic body radiation therapy for inoperable hepatocellular carcinoma as a local salvage treatment after incomplete transarterial chemoembolization. Cancer 2012 [published online ahead of print May 8 2012]. Accessed October 1, 2012 10.1002/cncr.27533 22570179

[B9] O’Connor JK Trotter J Davis GL Long-term outcomes of stereotactic body radiation therapy in the treatment of hepatocellular cancer as a bridge to transplantation Liver Transpl 2012 18 949 954 10.1002/lt.23439 22467602

[B10] Huang W-Y Jen Y-M Lee M-S Stereotactic body radiation therapy in recurrent hepatocellular carcinoma Int J Radiat Oncol Biol Phys 2012 84 355 361 10.1016/j.ijrobp.2011.11.058 22342300

[B11] Kopek N Holt MI Hansen AT Høyer M Stereotactic body radiotherapy for unresectable cholangiocarcinoma Radiother Oncol 2010 94 47 52 10.1016/j.radonc.2009.11.004 19963295

[B12] Barney BM Olivier KR Miller RC Haddock MG Clinical outcomes and toxicity using Stereotactic Body Radiotherapy (SBRT) for advanced cholangiocarcinoma Radiat Oncol 2012 7 67 10.1186/1748-717X-7-67 22553982 PMC3464963

[B13] Herfarth KK Debus J Lohr F Stereotactic single-dose radiation therapy of liver tumors: results of a phase I/II trial J Clin Oncol 2001 19 164 170 11134209 10.1200/JCO.2001.19.1.164

[B14] Herfarth KK Debus J Stereotactic radiation therapy for liver metastases Chirurg 2005 76 564 569 10.1007/s00104-005-1039-5 15877214

[B15] Rusthoven KE Kavanagh BD Cardenes H Multi-institutional phase I/II trial of stereotactic body radiation therapy for liver metastases J Clin Oncol 2009 27 1572 1578 10.1200/JCO.2008.19.6329 19255321

[B16] McCammon R Schefter TE Gaspar LE Observation of a dose-control relationship for lung and liver tumors after stereotactic body radiation therapy Int J Radiat Oncol Biol Phys 2009 73 112 118 10.1016/j.ijrobp.2008.03.062 18786780

[B17] Dawson LA McGinn CJ Normolle D Escalated focal liver radiation and concurrent hepatic artery fluorodeoxyuridine for unresectable intrahepatic malignancies J Clin Oncol 2000 18 2210 2218 10829040 10.1200/JCO.2000.18.11.2210

[B18] Park HC Seong J Han KH Dose–response relationship in local radiotherapy for hepatocellular carcinoma Int J Radiat Oncol Biol Phys 2002 54 150 155 12182985 10.1016/s0360-3016(02)02864-x

[B19] Wada H Takai Y Nemoto K Yamada S Univariate analysis of factors correlated with tumor control probability of three-dimensional conformal hypofractionated high-dose radiotherapy for small pulmonary or hepatic tumors Int J Radiat Oncol Biol Phys 2004 58 1114 1120 10.1016/j.ijrobp.2003.08.012 15001252

[B20] Blomgren H Lax I Näslund I Svanström R Stereotactic high dose fraction radiation therapy of extracranial tumors using an accelerator: clinical experience of the first thirty-one patients Acta Oncol 1995 34 861 870 10.3109/02841869509127197 7576756

[B21] Schefter TE Kavanagh BD Timmerman RD A phase I trial of stereotactic body radiation therapy (SBRT) for liver metastases Int J Radiat Oncol Biol Phys 2005 62 1371 1378 10.1016/j.ijrobp.2005.01.002 16029795

[B22] Méndez Romero A Wunderink W Hussain SM Stereotactic body radiation therapy for primary and metastatic liver tumors: A single institution phase i-ii study Acta Oncol 2006 45 831 837 10.1080/02841860600897934 16982547

[B23] Hoyer M Roed H Traberg Hansen A Phase II study on stereotactic body radiotherapy of colorectal metastases Acta Oncol 2006 45 823 830 10.1080/02841860600904854 16982546

[B24] Kavanagh BD Schefter TE Cardenes HR Interim analysis of a prospective phase I/II trial of SBRT for liver metastases Acta Oncol 2006 45 848 855 10.1080/02841860600904870 16982549

[B25] Katz AW Carey-Sampson M Muhs AG Hypofractionated stereotactic body radiation therapy (SBRT) for limited hepatic metastases Int J Radiat Oncol Biol Phys 2007 67 793 798 10.1016/j.ijrobp.2006.10.025 17197128

[B26] Tse RV Hawkins M Lockwood G Phase I study of individualized stereotactic body radiotherapy for hepatocellular carcinoma and intrahepatic cholangiocarcinoma J Clin Oncol 2008 26 657 664 10.1200/JCO.2007.14.3529 18172187

[B27] Lee MT Kim JJ Dinniwell R Phase I study of individualized stereotactic body radiotherapy of liver metastases J Clin Oncol 2009 27 1585 1591 10.1200/JCO.2008.20.0600 19255313

[B28] Cárdenes HR Price TR Perkins SM Phase I feasibility trial of stereotactic body radiation therapy for primary hepatocellular carcinoma Clin Transl Oncol 2010 12 218 225 10.1007/s12094-010-0492-x 20231127

